# Antibiotics prescription habits of Spanish endodontists: Impact of the ESE awareness campaign and position statement

**DOI:** 10.4317/jced.59053

**Published:** 2022-01-01

**Authors:** Alfonso López-Marrufo-Medina, Laura Domínguez-Domínguez, Daniel Cabanillas-Balsera, Victoria Areal-Quecuty, Isabel Crespo-Gallardo, Mª Carmen Jiménez-Sánchez, José López-López, Juan J. Segura-Egea, Jenifer Martin-Gonzalez

**Affiliations:** 1 Endodontic Section, Department of Stomatology, University of Sevilla, C/Avicena s/n, 41009 Sevilla, Spain; 2 Department of Odontostomatology, Faculty of Medicine and Health Sciences (Dentistry), Barcelona University Dental Hospital, University of Barcelona, 08970 Barcelona, Spain

## Abstract

**Background:**

The inadequate use of antibiotics by dentists can contribute to antibiotic resistance. The European Society of Endodontology (ESE) has published a scientific evidence-based position on antibiotic use in endodontic infec-tions. The aim of this study was to analyze the antibiotics prescription habits of Spanish endodontists in the management of endodontic infections, comparing them with those they had 10 years ago, to assess the impact of the ESE awareness campaign and position statement on antibiotics in endodontics.

**Material and Methods:**

One hundred Spanish endodontists were requested to answer to a one-page survey, similar to that used previously ten years ago in another study, on indications for systemic antibiotics in the management of endodontic infections. Data were analyzed using descriptive statistics and chi-square test. Seventy-seven endodontists (77%) completed satisfactorily the survey and were included in the study.

**Results:**

The average duration of antibiotic therapy was 5.64 ± 1.75 days. In patients with no medical allergies, 97.1% selected amoxicillin as the first-choice antibiotic. The first drug of choice for patients with an allergy to penicillin, was clindamycin 300 mg (74.03%). For cases of pulp necrosis with asymptomatic apical periodontitis, fistulous tract and mild/symptomatic symptoms, 100% of endodontists would prescribe antibiotics. For the scenario of a pulp necrosis with symptomatic periodontitis apical and no swelling, 20% endodontists would prescribe antibiotics.

**Conclusions:**

Antibiotics prescription habits of Spanish endodontists has improved after the ESE awareness campaign and position statement on antibiotics in endodontics. Even so, there are a percentage of professionals that still prescribe antibiotics erro-neously.

** Key words:**Antibiotic, antibiotics resistance, dentistry, endodontists, endodontics, prescription habits, primary care

## Introduction

The main objective of root canal treatment is to prevent or cure apical periodontitis (1). Pulp and periapical pathology is caused by bacteria and their virulence factors (2). Most of these inflammatory-infectious conditions have a short duration and easy resolution as long as they are diagnosed early and the etiology factor is treated or removed through root canal treatment. However, there are some pulpal or periapical conditions, as well as patients, where the administration of antibiotics is necessary, in addition to endodontic therapy (3).

Endodontic infections are polymicrobial, involving a combination of gram-positive, gram-negative, facultative anaerobes and strict anaerobic bacteria. Usually, bacterias are usually organized in biofilm communities and can colonize the main canal and also other areas in the root canal system (4). In primary endodontic infection, that occurs in root canals that have previously not been treated, is frecuently polymicrobial in nature with gramnegative and gram-positive bacteria, dominated by obligate anaerobes. The persistent/secondary endodontics infections are typically more complex mixed-species, where gram-negative bacteria are the most dominant. Nowdays, a significantly greater microbial diversity has been demonstrated in both type of infections. *Prevotella*, *Fusobacterium*, *Porphyromonas*, *Parvimonas*, and *Streptococcus* are commonly detected genera in both type of infections. *Enterococcus faecalis* is frequently associated with treatment failure (5-8). *Firmicutes*, *Bacteroidetes*, *Proteobacteria*, *Actinobacteria*, and *Fusobacteria* are the most abundant phyla, regardless of the infection type. Futhermore, archaea (9), viruses (10) and fungi, with *C. albicans* the most frecuently, are also implicated in endodontic disease (11).

In determinated clinical situations produced by endodontic infections, systemic antibiotic treatment in conjunction with endodontic therapy is indicated in. Beta-lactam antibiotics (penicillin V and amoxicillin) are recommended for the treatment of endodontic infections. Recommended loading doses are 1000 mg of penicillin V administered orally followed by 500 mg every 4–6 h or 1000 mg amoxicillin, with or without clavulanic acid, followed by 500 mg every 8 h. If penicillin V is used and therapy is ineffective, the combination of penicillin V with metronidazole (loading dose 1000 mg followed by 500 mg every 6 h) or amoxicillin with clavulanic acid is recommended. In patients with penicillin allergy, clindamycin (600 mg loading dose followed by300 mg every 6 h), clarithromycin (500 mg loading dose followed by 250 mg every 12 h) or azithromycin (loading dose of 500 mg followed by 250 mgonce a day) can be prescribed. As part of general antibiotic stewardship, it is currently recommended to prescribe antibiotics for 3 days and review the patient; further antibiotics should only be pre-scribed if indicated clinically (3,12-14).

Systemic antibiotics should be prescribed at the correct frequency, dose and duration so that the minimal inhibitory concentration is surpassed and so that side effects and the selection of resistant bacteria are prevented (15). Since Alexander Fleming discovered penicillin in 1928 and Florey introduced it to the clinical practice in 1940, antibiotics have been increasingly abused in Dentistry (16). The abusive use of antibiotics has triggered an immunity of the bacteria to some of them, producing the so-called antibiotic resistances (17). Antibiotic resistance is the ability of a microorganism to resist the effects of such drugs and is produced by genetic changes in bacteria that are highly exposed to the drug (18). This exaggerated exposure has led to a decrease in the sensitivity of the bacteria found in the oral cavity, specifically those that have developed the greatest resistance are *Porphyromonas* spp and *Prevotella* spp. In this way, antibiotic resistance has become a serious problem for world public health, a problem in which dentists, and especially endodontists, have a big responsibility due to the high prevalence of endodontic infections (12).

Different studies carried out on endodontists (17), postgraduate students of endodontics (19), under-graduate students of dentistry (20) and surgeons (21) all in Spain as well as studies carried out in the USA (22) and different European countries (23-25) reveal the inadequate use of antibiotics by general dentists and endodontists worldwide. This is due to the lack of knowledge about pharmacology, commercial and social pressure on the use of these drugs by dentists worldwide (19).

In order to improve this impending problem, scientific guidance based on scientific evidence is established by a committee of experts from the European Society of Endodontology (ESE) in 2018 (3).The key feature of ESE is to emphasize the appropriate use of antibiotics in endodontics and the need to place more emphasis on the performance of root canal treatment exclusively. In particular, ESE places value to the risks associated with the inappropriate use of antibiotics and especially antibiotic resistance. To this end, ESE hopes that all national societies will develop a national awareness through campaigns that reach out to dental professionals, other health professionals, other national medical and dental societies and the public. This campaign, which began in November 2018, includes the publication of scientific evidence-based position on antibiotic use. In Spain, Endodoncia, the official journal of the Spanish Endodontic Society (AEDE), has given prominence to this campaign and the criteria established by the ESE through publications to raise awareness among Spanish general dentists and endodontists. In 2021, it has been three years since the publication of these position, which makes it possible to assess the impact they have had on the clinical practice of endodontists.

The aim of this study was to analyze the antibiotics prescription habits of Spanish endodontists in the management of endodontic infections, comparing them with those they had 10 years ago, to assess the impact of the European Society of Endodontology (ESE) awareness campaign and position statement on antibiotics in Spain.

## Material and Methods

-Study Population

In this cross-sectional survey, 100 Spanish endodontists were asked to respond to a survey on indications for systemic antibiotics in the treatment of endodontic infections and antibiotic prophylaxis.

-Questionnaire

**Figure 1:**
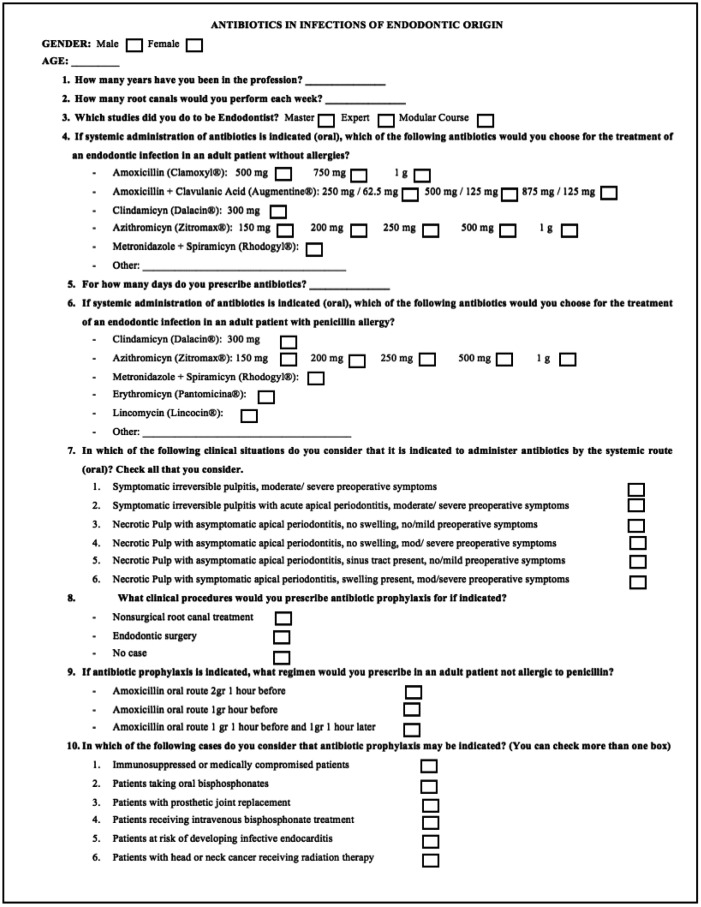
Survey on antibiotic prescribing habits in the treatment of endodontic infections.

The questions included in the survey (Fig. 1) and the different endodontic situations proposed were based on surveys from previous published studies, in the United States (26,27) and Spain (10).

-Data Collection and Statistical Analysis

For the collection of the data, the Excel program version 15.40 (Microsoft Corp., Redmond, WA, USA) was used, describing them by means of frequency Tables. The numerical variables are expressed as mean ± standard deviation. The data were analyzed using descriptive statistics and the chi-square test. Significant differences were considered when *p* < 0.05.

## Results

-Participation and Description of Respondents

The sample consisted of a total of 41 women (53.3%) and 36 men (46.8%). The mean age of the respondents was 36.2 ± 9.9 years. The average clinical experience of the respondents was 11.6 ± 8.7 years. The respondents were classified high and low experience (more or less than 75% of the average experience of the respondents, respectively), being more prevalent low experience (n=42, 54.5%). The number of weekly root canals were 16.3 ± 9.3. In regard to the specialization of endodontists, in 57 (61.3%) the Master’s degree in Endodontics was among their postgraduate studies, in 18 (19.4%) the title of Expert in Endodontics and in 18 (19.4%) some modular course in this field. The average age of post graduated education duration was 22.1 ± 9.5 months.

-Antibiotics prescription

In case of patients with no medical allergies, most of the respondents chose amoxicillin as first-choice antibiotic, alone (61.0%) or in combination with clavulanic acid (35.0%), 1.3% chose Clindamycin (Dalacin®) 300mg, another 1.3% chose Azithromycin (Zitromax®) 1g and another 1.3% chose Metronidazole + Spiramycin (Rhodogyl®) (Table 1).

**Table 1:**
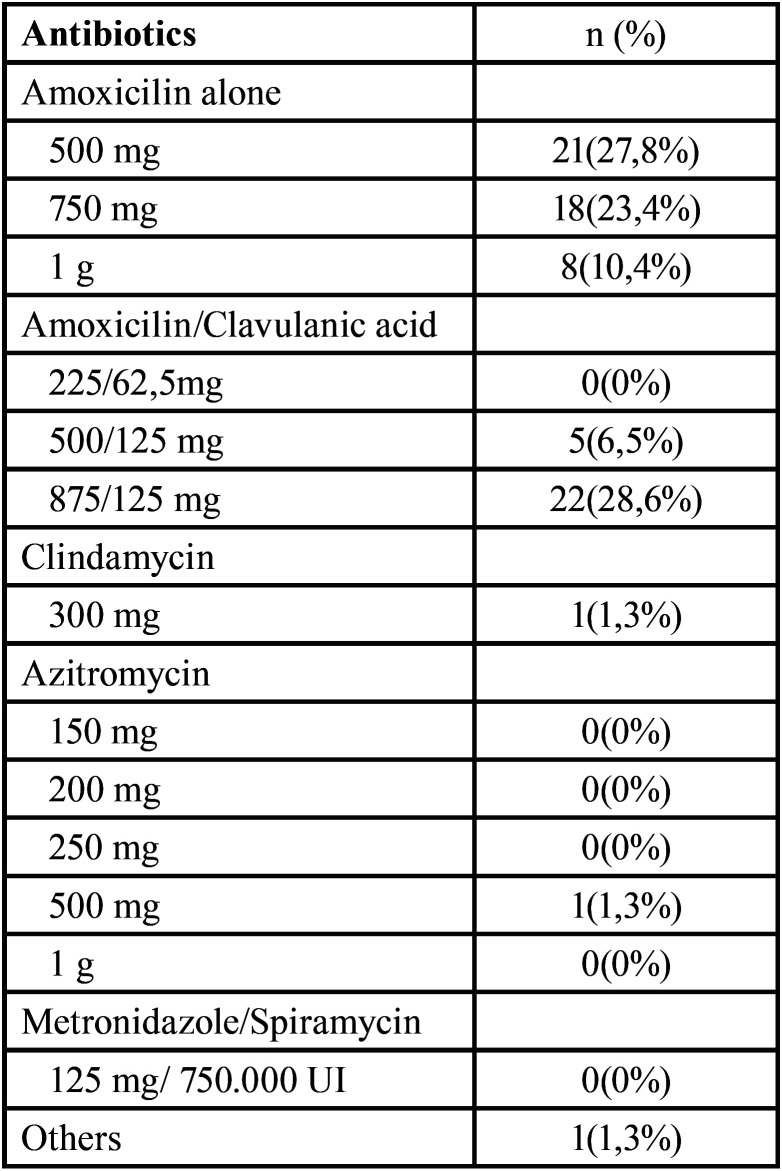
Antibiotic preference in patients with no medical allergies.

Regarding the influence of the experience in the type of antibiotic prescribed, endodontists with a longer professional career preferred amoxicillin 500 mg and those with less experience prescribed Augmentine 875/125 mg, but difference was not significant (*p* > 0.05). The type of post-graduated education program of the re-spondents also did not influence the choice of antibiotic. The respondents with master’s degrees preferred Augmentine 875/125mg, and the rest of participants choose amoxicillin 500 mg (*p*>0.05). In patients with medical allergies, most of the Spanish endodontists prescribed Clindamycin (Dalacin®) 300 mg (n=57, 74.03%) as first treatment option.

-Duration of Antibiotic Treatment

**Figure 2:**
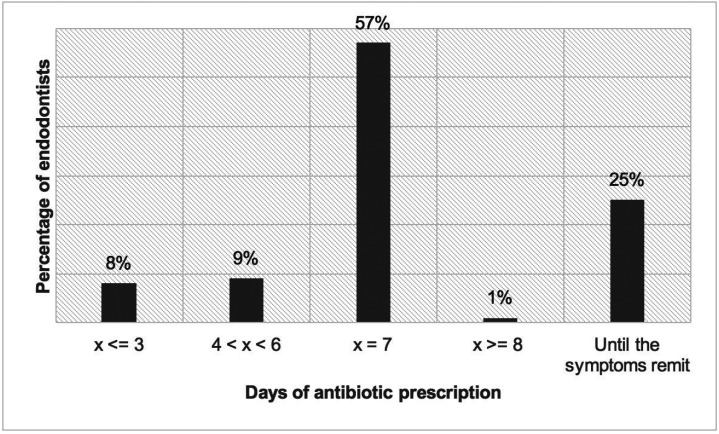
Distribution of responses by treatment duration.

The average duration proposed for antibiotic therapy was 5.6 ± 1.8 days. More than a half of respondents (56.8%) would prescribe antibiotics for 7 days and only 24.7% of endodontists prescribed until the sympto-matology remits (Fig. 2).

Endodontists with less clinical experience indicated antibiotics more frequently for 3 days or until symptoms remitted than those with more clinical experience (*p*>0.05). Depending on the type of post graduated education program, it was observed that professionals who had completed a master’s degree prescribed more the option “3 days or until symptoms remit” than those who had completed another post-graduate degree (*p*>0.05).

-Antibiotic prescription according to type of endodontic infection

It turned out that all the respondents (100%) prescribe antibiotics in case of necrotic pulp with symptomatic apical periodontitis, abscess and moderate/severe symptoms. Most of the Spanish endodontists (92%) agreed that it is not necessary to prescribe antibiotics for irreversible pulpitis with symptomatic apical periodontitis. Likewise, up to 12% of the respondents prescribe antibiotics in cases of necrotic pulp with asymptomatic apical periodontitis, fistulous tract and mild/symptomatic symptoms and 25% prescribe antibiotics in cases of necrotic pulp with apical periodontitis and no swelling.

When the patient’s diagnosis was necrotic pulp, asymptomatic apical periodontitis, no swelling and no or mild symptoms, endodontists who studied a master’s degree erroneously prescribed in this clinical condition less frequently than those who studied another postgraduate degree. Even so, the difference was not significant (*p*>0.05).

## Discussion

This is the first study investigating the possible influence of ESE position on antibiotics use on the pattern of antibiotic prescription by Spanish endodontists by comparing the results of the present study with previous studies published with a similar sample in this country.

The sample corresponded to Spanish dentists who had specialized in Endodontics. The questions that formed part of the survey and the different endodontic situations proposed were based on surveys from previous studies published in the United States (26,29) and Spain (21).

The response rate of the respondents was very high (77%) coinciding with other previous studies (28). The gender was mostly female (71%) according to Martínez-Beneyto *et al*. in 2012 (29). The clinical experience of the endodontists was 11.6 ± 8.7 years; the weekly number of root canals treatments was 16.3 ± 9.4; the percentage of the respondents who studied a master’s degree, modular course or expert course was 61.3%, 19.4% and 19.4% respectively and the average duration of the educational program was 22.1 ± 9.5 months. These data cannot be compared with other studies since this is the first time they have been taken into account.

In 2018 the protocol for the use of antibiotics is published by a committee of experts of the European Society of Endodontics (3). This protocol is used as reference to qualify as wrong or right the answers of the respondents in this study. With regard to the type of antibiotic prescribed by the respondents for patients with no allergies to penicillin, it was 96.1% amoxicilin (alone in 61% and in combination with clavulanic acid in 35.1%). This high percentage coincides with the study conducted by Rodríguez-Nuñez *et al*. on members of the Spanish Endo-dontics Society prior to publication of the guide, which was 86.1% (5). In the present study, the antibiotic of first choice was amoxicillin (alone or in combination with clavulanic acid) according to the majority of published papers (17,19-21). The remaining European countries also chose amoxicillin as the antibiotic of choice, either alone or in combination with clavulanic acid (12,23-25). In case of USA, it does not coincide with Spain and Europe, since in the published studies they chose penicillin to treat infections of endodontic origin (12). In relation to the type of postgraduate training undertaken to reach the specialty, we found that students of experts program and modular courses would prescribe more amoxicillin alone than those who studied masters. This could be due to the fact that, although the master’s degree is on average longer and involves greater specialization, the same hours are reserved for teaching on antibiotic prescription as in the case of other postgraduate studies.

In patients allergic to penicillins, both this study and previous studies, coincide with the scientific reference guide and it was correctly prescribed clindamycin 300 mg. The duration of antibiotic therapy was 5.6 ± 1.8 days. It had been shown that there are 56.78% of respondents who prescribe antibiotics for 7 days, followed by the option “until symptoms remit” with 24.7% of respondents. These data are encouraging since they do not coincide with the study conducted by Rodríguez-Núñez *et al*. before the publication of the guide, in which the duration of treatment amounted to 6.8 days ± 1.8 days and the frequency of prescription with duration of 7 days of antibiotic therapy was 64.2% (17). This may be due to the importance given in this specialty of Dentistry to the correct diagnosis of pulp and periapical pathology, training on antibiotics and antibiotic resistance problem, a growing problem that can be a great burden to society in the future. Even so, it is necessary to make more emphasis on this aspect, since despite having a clear and established protocol supported by scientific evidence that the duration of antibiotic prescription should last about 3 days and be reviewed to suspend it in the case of having ceased the symptoms, more than half of the respondents continue prescribing it for a week (3). The most experienced respondents, who had completed a master’s degree, achieved a better adaptation of the treatment time with the European Association of Endodontology (*p*<0.05).

**Figure 3:**
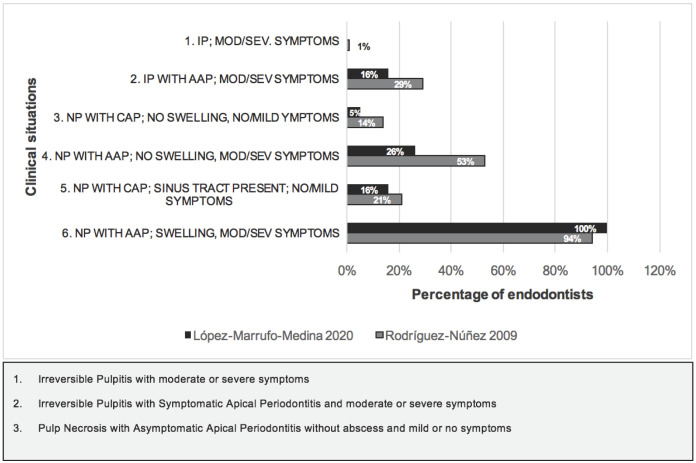
Antibiotic prescription in the 6 clinical situations of Rodríguez-Núñez *et al.* 2009 in comparison with the present study.

On the indications for antibiotics according to the pulp and periapical pathology diagnosed by the European Society of Endodontology (3), antibiotics must be prescribed when there is systemic involvement (fever >38ºC, general malaise, lymphadenopathies, trismus...), when we find a progression of the infection (increased in-flammation, cellulitis, osteomyelitis) or when the infection persists over time and in medically compromised patients (immunosuppressed). The present study shows lower percentages of endodontists prescribing anti-biotics in clinical situations that do not require them compared to previous similar studies in Spain (17,19-21) (Fig. 3). This improvement in prescribing habits could reveal the positive and direct effect that the awareness ESE campaign and position statement has had (3). In irreversible pulpitis and moderate/severe symptoms, none of the respondents proposed prescribing antibiotics, which was the right decision in this case. In 2009 Rodríguez-Núñez *et al*. recorded 11.4% of respondents who prescribed antibiotics unnecessarily (17). In the case of irreversible pulpitis with symptomatic apical periodontitis and moderate or severe symptoms, the wrong prescription of our respondents amounted to 16.39%, a similar Figure to that in the study conducted more than 10 years ago to Spanish endodontists (17), but also lower, since in this case 28.6% indicated antibiotic therapy wrongly. In cases of necrotic pulp with asymptomatic apical periodontitis with no swelling or symptoms, 5.19% prescribed antibiotics erroneously, again the trend of lesser error in this study is met compared to studies prior to the publication of the guide (3,17,19-21), which recorded 14.3% of endodontists prescribing erroneously. In the case of necrotic pulp, with symptomatic apical periodontitis without abscess and moderate and severe symptoms, it had been found the highest rate of antibiotic prescribed erroneously, both in our respondents and in the rest of the studies, although to a greater extent. It had been observed that 26% of them indicated as necessary antibiotic coverage, unlike the study of 2009 to Spanish endodontists (17) that 52.9% prescribed antibiotics in this clinical situation. In the case of differentiating them by their specialty, a greater error in the case of master students was found, with no difference statistically significant (*p*>0.05). In necrotic pulps with asymptomatic apical periodontitis, fistulous tract with mild symptoms or without symptoms the results follow the same pattern as in the rest of the indications. In 2009, 15.6% of the respondents prescribed wrongly but to a lesser extent than endo-dontists (17). Only in cases of necrotic pulp with symptomatic apical periodontitis, abscess and moderate and severe symptoms was antibiotic coverage necessary. One hundred percent of endodontists proposed adjuvant antibiotic therapy for root canal treatment. This Figure continues to be more positive than in the Rodriguez.

The results of the present study show that there has been an improvement in the prescribing habits of Spanish endodontists in the last decade, which may be due to the publication of scientific guides on this subject in recent years. Prescribing has improved, but there are still endodontists who prescribe incorrectly, so it is necessary and essential to develop new strategies to improve the knowledge and prescribing habits of endodontists in Spain in the treatment of pulp and periapical pathologies. This trend towards improvement without reaching absolute awareness coincides with other studies based on the impact of clinical practice guidelines such as Silva Conde *et al*. in 2019 (30). It is necessary to review and to improve the teaching of postgraduate educational programs in Endodontics, both master’s and expert or modular, as well as the teaching programs of the different professional associations of dentists and, of the congresses held by the scientific societies of the specialty on antibiotic pre-scription and patient management.

## Conclusions

The publication of a position statement based on scientific evidence has resulted in an improvement in the prescription of antibiotics by Spanish endodontists. However, although the pattern of prescribing has improved in the last decade it is still not completely in line with the scientific evidence.
